# Sea Anemones, Actinoporins, and Cholesterol

**DOI:** 10.3390/ijms23158771

**Published:** 2022-08-07

**Authors:** Juan Palacios-Ortega, Diego Heras-Márquez, Rafael Amigot-Sánchez, Carmen García-Montoya, Carlos Torrijos, Diego Laxalde, José G. Gavilanes, Sara García-Linares, Álvaro Martínez-del-Pozo

**Affiliations:** 1 Departamento de Bioquímica y Biología Molecular, Facultades de Ciencias Biológicas y Químicas, Universidad Complutense de Madrid, 28040 Madrid, Spain; 2 Biochemistry Department, Faculty of Science and Engineering, Åbo Akademi University, FI-20520 Turku, Finland

**Keywords:** pore-forming proteins, actinoporins, sticholysin, equinatoxin, fragaceatoxin, sphingomyelin, cholesterol

## Abstract

Spanish or Spanish-speaking scientists represent a remarkably populated group within the scientific community studying pore-forming proteins. Some of these scientists, ourselves included, focus on the study of actinoporins, a fascinating group of metamorphic pore-forming proteins produced within the venom of several sea anemones. These toxic proteins can spontaneously transit from a water-soluble fold to an integral membrane ensemble because they specifically recognize sphingomyelin in the membrane. Once they bind to the bilayer, they subsequently oligomerize into a pore that triggers cell-death by osmotic shock. In addition to sphingomyelin, some actinoporins are especially sensible to some other membrane components such as cholesterol. Our group from Universidad Complutense of Madrid has focused greatly on the role played by sterols in this water–membrane transition, a question which still remains only partially solved and constitutes the main core of the article below.

## 1. Historical Context

The Iberian Peninsula stands out in the field of protein-lipid interactions. Only a quick look into the Portuguese or Spanish Biophysics Societies member lists (https://www.spbf.pt/, https://sbe.es/) is needed to discover dozens of very well-known names in the field of membrane and protein biophysics, using an enormous variety of techniques and approaches, including a trademark going back 50 years, when the first seeds of these groups were sown.

Actinoporins began to be an important subject of study for some of these scientists when, at the end of the 20th century [1,2,3,4], a Cuban group led by María Eliana Lanio, Fabiola Pazos, and Carlos Álvarez began to spread the word, sending several of their postdocs and students to different labs in Europe [5,6]. In Spain, they came to the Biophysics Institute at Bilbao (https://www.biofisika.org/en), led by Félix Goñi and Alicia Alonso, and to our Protein Function and Structure Group (ESFUNPROT; https://www.ucm.es/grupoesfunprot/), funded and directed by José G. Gavilanes, one of the authors of this article.

The researcher who led the actinoporin research in Bilbao, Juan Manuel González Mañas, deserves special mention since his contribution was key to finally solving the only known three-dimensional structure of an actinoporin pore with atomic resolution [7,8,9,10,11,12,13,14].

This movement sparked an intricate web of interactions that comprises many diverse groups around the world. Restricting ourselves only to those groups led by Spanish scientists in the field of actinoporins, Ana Jesús García Sáez, presently working in the CECAD, University of Cologne in Germany (https://garcia-saez.cecad-labs.uni-koeln.de/group-leader, accessed on 10 July 2022), and José Manuel Martínez Caaveiro, who is now located at the Graduate School of Pharmaceutical Sciences in Kyushu University in Japan (https://www.isc.kyushu-u.ac.jp/graduate/voices/phar/, accessed on 10 July 2022), should also be named. García-Sáez has made seminal contributions in solving the mechanism of action of pore-forming proteins involved in triggering apoptosis [15], but has also enlightened us with new data regarding actinoporins’ function, in particular about the existence of different stoichiometries, not only along the pore-formation pathway but also when thermodynamically stable pores are finally established [16,17,18]. Martínez-Caaveiro, former Ph. D. student of the Bilbao group, was the lead scientist in the research that solved the already mentioned three-dimensional structure of the Fragaceatoxin C (FraC) membrane pore [14].

Finally, Mercedes Ferreras, who graduated from ESFUNPROT in the early 1990s, was one of the first scientists to perform electrophysiological measurements involving the actinoporins sticholysins, the main actors of this article. A work she performed as a member of the group led by the late, and highly appreciated, Gianfranco Menestrina in Trento (Italy) [19].

All these groups, which share Spanish roots, have made seminal contributions to the establishment of what we now know about the behavior of actinoporins [10,14,16,17,20,21,22,23,24,25]. Several other groups and scientists have also made key discoveries regarding actinoporins’ structure and function. They are not specifically mentioned in this paragraph simply because they do not have those Spanish roots, but we think that their work is largely recognized across the article, as can be confirmed from examining the list of references at the end. We hope we did not forget anybody, but apologize in advance in case we did.

## 2. Sea Anemones Actinoporins

**Figure 1:**
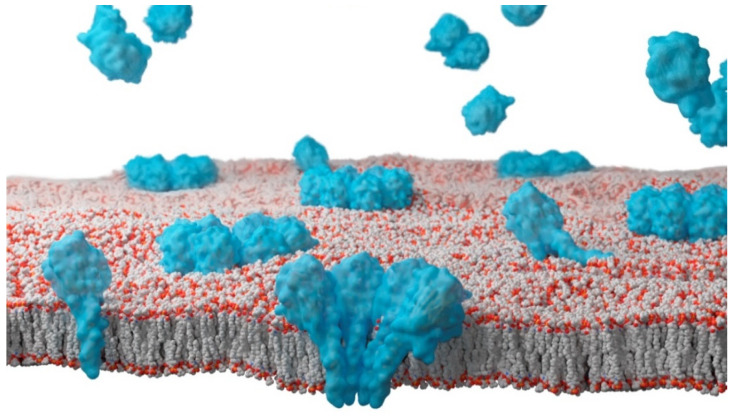
Artistic rendering of the process of pore formation by sticholysins. Lipids in grey (carbon atoms), red (oxygen atoms), and orange (phosphorus atoms). Actinoporins in light blue. Water-soluble monomers, and possibly dimers, (top, with no contact with the membrane) [14,24,48,49] would bind the membrane as monomers or higher-order oligomers. The helix would then be extended and deployed, lying on the membrane surface. Then, it would penetrate the bilayer, disrupting membrane continuity. The order in which all these steps take place, the stoichiometry of the different intermediates, and the existence, or not, of prepore complexes, is still matter of discussion. Finally, the thermodynamically stable pore complexes would assemble into octameric pores (at the front of the figure it is shown a cross-section of a final pore to illustrate its inner arrangement). This figure was generated using UCSF Chimera, UCSF ChimeraX (https://www.cgl.ucsf.edu/chimerax/docs/credits.html), and composed and rendered in Blender (Community, B. O. (2018). Blender—a 3D modelling and rendering package. Stichting Blender Foundation, Amsterdam. Retrieved from http://www.blender.org).

Venoms have lately acquired a great deal of attention [26,27]. Developments in omics fields have greatly facilitated their study and classification [28]. Many scientific groups work not only in systematically finding new compounds and substances, with sometimes unexpected or interesting properties, but also in transforming those molecules into useful, safe and bio-sustainable biotechnological tools [29,30,31,32], including many with potential therapeutic uses [26,33]. Attention began to focus on coelenterates venom as early as the 1960s [34,35,36]. This was rather late, however, considering that jellyfishes and sea anemones have been known to be toxic since ancient times [37,38]. Eventually, this research led to the discovery of actinoporins, cytolytic pore-forming proteins found in the venom of several species of sea anemones [39,40,41,42,43,44,45,46]. Their most remarkable ability, and the basis for their function, is their capacity to metamorphose between two different folds. One is water-soluble, whereas the other one, triggered by its encounter with a membrane containing sphingomyelin (SM), leads to membrane binding and oligomerization. This process results in the actinoporin monomers assembling into an oligomeric transmembrane pore complex (Figure 1). It is a transformation that takes place in the absence of any chemical modification involving the formation or destruction of a covalent bond, neither the coupling to any other external source of energy such as an ATP hydrolysis reaction, for example. This is a striking result showing, in black and white, the importance of the environment in protein folding, as envisioned by Anfinsen in the 1960s [47]. Final assembly of these pores produces cell death by osmotic shock [42,43,44,45].

However, the physiological role of actinoporins is not really known yet, aside from a general idea that they can function as part of both defensive and predatory mechanisms. Even the identity of their natural targets remains unknown, although it is assumed that they are mainly “aimed” towards some crustaceans or small fish. Some authors even speculate that, in addition to producing an osmotic shock, the pore would also act as a channel to deliver some other small components of sea anemones’ venom into the cells. Given the presence of fenestrations within the pore lumen, it is also possible to consider that some of these substances might be delivered into the hydrophobic environment of the membrane bilayer [14].

The most studied sea anemone actinoporins are sticholysins (Stns), produced by *Stichodactyla helianthus* [19,50,51,52], equinatoxins (Eqts), from *Actinia equina* [53,54], and fragaceatoxins (Fras), from *Actinia fragacea* [12,13,14,24,55]. StnII was in fact one of the first actinoporins discovered when it was reported the specific binding of a *S. helianthus* (then *Stoichactis helianthus*) toxin fraction to SM [56,57]. Those scientists even obtained the first micrographs of an actinoporin pore structure, and this happened as early as 1977 [58]. Nowadays, we already know that at least 20 distinct species of sea anemones produce proteins of this family [12,39,40,59].

All actinoporins show remarkably similar amino acid sequences, in most cases sharing more than 60% of sequence identity [41,42,45]. This similarity is especially important in the case of isoforms (toxins with very similar activity and molecular mechanism of action, but different amino acid sequence and coded by different genes) produced by the same sea anemone species, which many individuals can synthesize simultaneously. Actinoporins such as StnI and StnII, for example, show 93.7% of sequence identity but their hemolytic potency is one order of magnitude different, and show quite distinct sensitivity regarding their behavior against Chol [60]. This group of toxic proteins are hence considered to constitute multigene families [14,39,40,59,61,62,63]. A molecular diversity that, within a single individual, seems to be helpful in broadening the range of accessible targets [14,22,23]. However, this observation is not well understood yet, since despite having multiple actinoporin sequences encoded in their genome, most of them generally produce only a small number of these toxins in detectable amounts [39,40,51,62,64,65].

**Figure 2:**
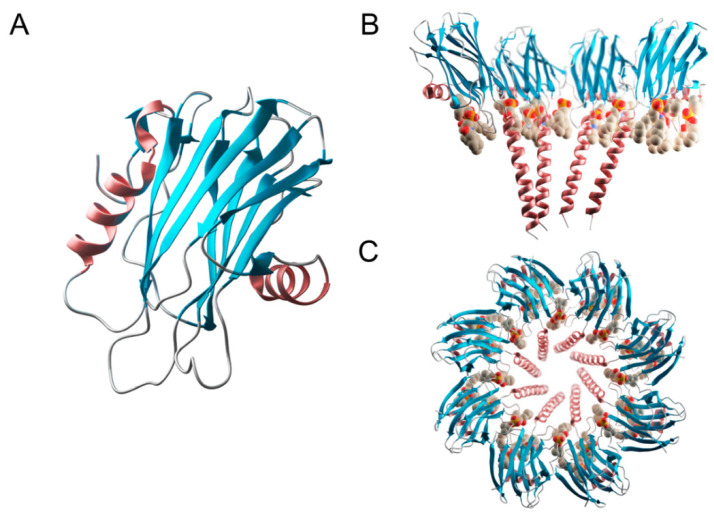
Three-dimensional structure of FraC. (**A**) Structure of a monomer of FraC in its soluble state (PDB ID: 3VWI). (**B**) Side-view of the octameric FraC pore (PDB ID: 4TSY). Only four monomers are displayed, to show the lumen of the pore. The lipids (in tan) can be seen exposed to the lumen in between the helices. (**C**) Top view of the octameric FraC pore. This figure was generated using UCSF Chimera, UCSF ChimeraX (https://www.cgl.ucsf.edu/chimerax/docs/credits.html), and composed and rendered in Blender (Community, B. O. (2018). Blender—a 3D modelling and rendering package. Stichting Blender Foundation, Amsterdam. Retrieved from http://www.blender.org).

As mentioned above, at least 20 different species of sea anemones have been observed to produce actinoporins, though only four of them have been studied in deep detail (StnI, StnII, EqtII, and FraC) [12,39,40,45,59]. Despite this rather wide distribution, and in agreement with their high degree of sequence identity, all actinoporins seem to share three-dimensional structures, both water-soluble and membrane-bound [14,55,66,67,68,69]. Only the water-soluble structures of five actinoporins (the four just mentioned and FraE) have been solved in atomic detail [14,55,66,67,68,69,70]. All of them show a common fold consisting of a β-sandwich flanked by two α-helices, one of them at the N-terminal end. In fact, their first 30 residues fold into a short β-strand, followed by a 3_10_ helix and a 10-residue-long stretch constituting the N-terminal α-helix [14,66,67,68,69,70,71,72] (Figure 2). The 3_10_ helix is a type of secondary structure found in proteins that is the fourth most common type observed, following α-helices, β-sheets and turns. It constitutes nearly 10–15% of all helixes in protein’s secondary structures and is typically observed as an extension of α-helixes found at either their N- or C-termini, as seems to be precisely the case for actinoporins (Figure 2). Max Perutz, the renowned scientist who unveiled the three-dimensional structure of hemoglobin, wrote the first paper documenting its existence [73].

When encountering a suitable membrane, this 30-residue-long N-terminal stretch detaches from the β-sandwich and extends into a longer amphipathic α-helix, which is going to line the lumen of the final pore [10,71,74,75,76] (Figure 1 and Figure 2). Quite surprisingly, these first 30 residues contain most of the variability among these proteins, an observation that has been attributed to their different pore-forming ability, especially when analyzed in quantitative terms.

Along this article we mostly focus on StnI and StnII, the actinoporins on which this Spanish group has centered its attention for the last 20 years. Indeed, most of the studies considering the influence of Chol on actinoporins’ behavior have been made using these two Stns as a reference model [42,45,77,78,79,80,81,82,83].

Lipids, particularly SM, are the only elements that actinoporins require to bind to a membrane. This has been clearly shown using model membranes that lack non-lipid components such as proteins or sugars. Within this same idea, preincubation with SM, or its removal from erythrocytes with sphingomyelinase, inhibits their toxic action [19,50,57,58,60,84,85,86,87,88,89]. Binding to this sphingolipid is so specific that it is assumed that SM functions as a true membrane receptor for actinoporins and they are actually classified as such by their ability to bind specifically to SM. So far, no specific interaction of actinoporins with other types of sphingolipids has been described. On the contrary, it seems that sea anemones producing actinoporins do not show SM in their membranes but phosphonosphingolipids, which are structural analogs of SM. This observation has been interpreted as a form of self-protection against these components of their own venom [90]. Having said that, is also true that many other factors affecting membrane biophysics and introducing different geometries or degrees of accessibility can also have profound influence on actinoporins’ behavior when encountering the membrane. These differences, however, are mostly quantitative and do not necessarily imply a significantly different mechanism of action. For example, it has been shown that membrane phase coexistence, in the presence of cholesterol (Chol), changes in fluidity or bilayer compactness, and the intricacy of the interfacial hydrogen bonding network of SM can affect their activity [8,11,50,77,78,88,91,92,93,94]. Actually, membrane thickness can even influence actinoporins’ efficacy, apparently because of the fixed length of the helical stretch crossing the bilayer [80]. This observation seems to correlate with the thickness of the cell membranes of their assumed potential prey [80].

Regarding their behavior in absence of membranes, our experience shows that while StnI seems to be more prone to oligomerization in solution than StnII, a small percentage of StnII in StnI-StnII mixtures promotes oligomerization [49]. That is to say, StnII favors dimer formation in StnI-StnII mixtures beyond what is observed for StnI alone, even at ratios in which StnII is, by far, the minority component of the mixture. This most probably explains the synergistic interaction described for both proteins when encountering proper biological or model membranes [95], and could be the reason why the same species of sea anemone produces different isoforms. It would be a mechanism to not only enlarge the range of potential prey but also to modulate the potency of their cytolytic toxin action.

## 3. The Role of Cholesterol

Chol is a major component of animal plasma membranes. It is a highly hydrophobic lipid with profound implications in membrane biophysics. Given its absence in plants and most microorganisms, it is also the target of many toxins from unicellular organisms. When comparing the results of actinoporins assayed on model membranes, containing or not containing Chol, great differences in performance are observed. Understanding the effect of Chol on actinoporin behavior, and the biophysics behind it, is thus highly interesting.

Quite early, our group showed that, even in the absence of SM, StnII can be forced to produce leakage of the aqueous contents of model lipid vesicles composed of phosphatidylcholine (PC) if 20–25% of Chol was present [50]. Much has been learnt since then but, quite unexpectedly, the role played by this sterol in the actinoporin–membrane interaction is not completely understood yet.

One of the first observations suggesting a key role for Chol in actinoporins’ action was obtained when cell permeabilization was impaired by the preincubation of mammalian cells with the Chol-sequestering molecule cyclodextrin [92]. Years later, StnII variants with distinct abilities to interact with model membranes in the presence, or not, of Chol, revealed that this lipid is not only an important partner for SM, but that it also facilitates the structural changes required to make a pore [89]. Almost simultaneously, using either SM or dihydro-SM (lacking the *trans* Δ^4^ double bond of the long-chain base), we observed that whereas both StnI and StnII formed pores in unilamelar vesicles containing palmitoyl-SM (PSM) or oleoyl-SM (OSM), the toxins failed to similarly form pores in vesicles prepared from dihydro-PSM or dihydro-OSM. These SM variants were chosen because of their quite different hydrogen-bonding properties [77]. Then, we decided to try benzyl alcohol, a small hydrogen-bonding disrupting compound with affinity to lipid bilayer interfaces and discovered that it facilitated StnII-induced pore formation in dihydro-OSM bilayers [77]. Altogether, these results were interpreted as the hydrogen bonding network in the interfacial region preventing StnII from membrane binding and hence affecting their pore forming capacity. Quite at the same time, we also firmly established that pore formation by Stns could be enhanced by the presence of different sterols, regardless of their domain-forming capability [78,96]. These experiments showed then that the capability of the 3β-OH sterol to act as a hydrogen-bonding acceptor, together with the overall Chol effect of increasing membrane fluidity, were responsible for enhancing Stn-induced release of contents from large unilamelar model vesicles, without a concomitant order increasing of the SM phase [77,78,96]. These results were indeed consistent with many different observations, by us and other groups, that actinoporins preferentially bind at domain boundaries [20,22,89,92,94,96,97,98,99]. These fluid and more disordered membrane regions are richer in imperfections than the more ordered phases. The preferred interpretation is that these imperfections facilitate membrane penetration, while also increasing the local toxin concentration, overall reducing the energy barrier of the penetration step [8]. Furthermore, SM head groups at domain boundaries would be further exposed to the solvent, easing the recognition process. Chol would be responsible for many of these effects by promoting phase separation and by its preference to associate with SM within the membranes [100,101,102,103]. However, it was also quite clear that StnII is more affected by the presence of Chol than the other four more studied actinoporins [60,89,104], a conundrum that we have not been able to completely explain yet.

**Figure 3:**
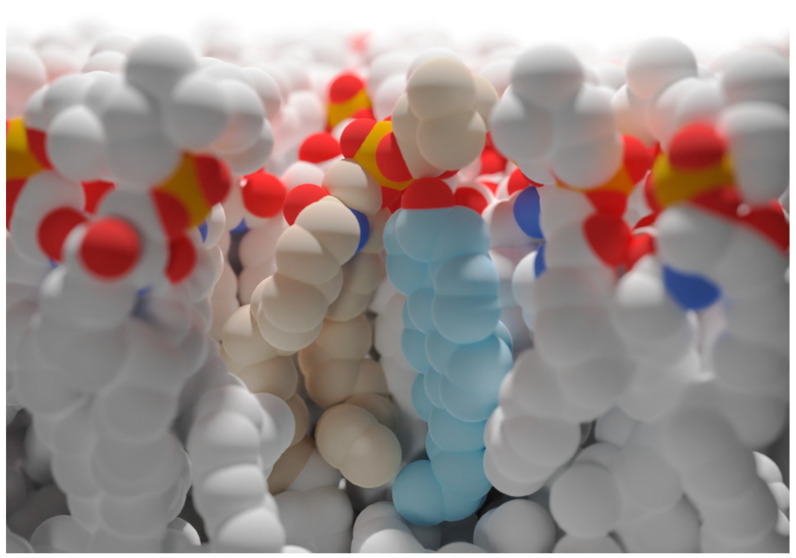
Artistic representation of the umbrella hypothesis. According to this hypothesis, Chol (in light blue) would be shielded from the solvent by the headgroup of neighboring SM molecules, such as the one in tan in the figure. For actinoporins, this could result in an easier recognition of SM. This figure was generated using UCSF Chimera, UCSF ChimeraX (https://www.cgl.ucsf.edu/chimerax/docs/credits.html), and composed and rendered in Blender (Community, B. O. (2018). Blender—a 3D modelling and rendering package. Stichting Blender Foundation, Amsterdam. Retrieved from http://www.blender.org).

It is known that Chol can modify the orientation and dynamics of the SM head group, as explained by the “umbrella hypothesis” [103,105,106,107] (Figure 3). According to this hypothesis, the sphingophospholipid headgroup is assumed to shield the hydrophobic region of cholesterol from unfavorable interactions with water [108]. This could be helpful for actinoporins when it comes to SM recognition [8]. It could also be also part of the explanation behind the aforementioned hydrogen-bonding network disrupting effect described in the previous paragraph. In fact, a few years ago Ostreolysin A (OlyA) was used to show that sphingomyelin adopts two distinct conformations in membranes when cholesterol is present [109]. One conformation appears bound to OlyA and is induced by stoichiometric association with Chol. The second one is free from Chol and does not bind to the protein. Quite strikingly, this ability to distinguish between free and Chol-bound SM was narrowed to a single Glu residue [109]. OlyA is another pore-forming protein, produced by the edible oyster mushroom (*Pleurotus ostreatus*), an organism not closely related to sea anemones, and is lytic to membranes containing *both* Chol and SM [110]. In light of the high structural similarity between OlyA and actinoporins [109], and considering the results reported in that article, we decided to start experiments, still ongoing, to explore if this is also the case for sticholysins. As of now, however, we can only speculate that engagement to SM might not be so similar as to OlyA, since this protein needs both lipids, SM and Chol, to exert its function, while actinoporins are still functional in the absence of this sterol (see for example [60]).

Although it is not yet understood in sufficient detail, what has been unequivocally demonstrated is that including a small percentage of Chol significantly increases StnII-induced calcein release, while the acyl chain order of SM only increases modestly [88]. Chol-induced increased lipid packing is a consequence of its interactions with the acyl chains of co-lipids, affecting membrane fluidity [107,111]. Due to Chol’s preference for SM, it will mostly affect the acyl chain order of SM when included in a model membrane made of PC-SM. Consequently, it will also affect the SM hydrogen-bonding network and the overall properties of the SM-rich phase [112,113,114], as stated above. Work performed by the group led by the Spanish scientist José Manuel Martínez Caaveiro, already mentioned in the first section of this article, showed how a single residue at the N-terminal α-helix of FraC (Phe16, equivalent to Phe14 in StnII) is essential for pore formation of Fra C in Chol-rich membranes, with its substitution resulting in mutants whose ability to induce calcein release is nearly abolished [22]. In this same set of experiments [89], we also employed a StnII Ala10 mutant to study the influence of Chol on the helix-deploying activity of this actinoporin. Ala10, which was substituted by a Pro residue, is located at the edge of the region thought to become α-helical to form the pore [56]. Thus, this mutation, which is far away from the membrane-binding surface, has been shown to provide enough conformational stiffness so as to hamper the required N-terminal α helix extension completely [10,76,115] (Figure 1 and Figure 2). As expected, given that the β-core sandwich motif had not been altered, binding of StnII A10P was not substantially affected by the presence, or not, of Chol. However, calcein leakage activity in the absence of this sterol was diminished, confirming that pore formation, but not membrane recognition, was affected in this mutant. This result can now be explained by the much later observation that calcein leakage experiments do not really reflect the presence of thermodynamically stable and final pore structures but just the appearance of transient permeation events of the membrane while evolving to form those stable assembled pores [82].

A later study has showed that the overall structure of Chol, and not only its hydroxyl headgroup, is responsible for facilitating SM-recognition by StnII [81], as it might seem from the information in the previous paragraphs. Quite unexpectedly for us, oleoyl-ceramide, which also interacts with SM and has an equivalent interfacial hydroxyl moiety, did not promote the permeabilizing capabilities of StnII, nor promoted SM-acyl chain separation as Chol did, for example. Furthermore, it was also shown that StnII-binding to membranes containing both SM and Chol induced a rearrangement of these lipids in the bilayer, increasing the overall separation of the acyl chains of SM, while was also effective in extracting Chol from the PC-rich phase of the membrane [81]. Using Förster resonance energy transfer between Trp residues in StnII and a fluorescent analog of Chol, cholestatrienol, we could show that resonance energy transfer efficiency was highest between Trp residues in positions 110 and 114 of StnII and colestatrienol, compared to the three other Trp residues, which are further away from the bilayer-binding region of StnII. Trp 110 and 114 are two amino acid residues whose side-chain hydrophobicity we, and others, have shown to be extremely important for actinoporins binding to the membrane [104,116]. This approach also revealed that colestatrienol was preferentially distributed near StnII when equilibrium was reached [81].

As stated above, membrane thickness can also affect pore formation by actinoporins [80]. Chol generally increases membrane thickness. Neutron reflection studies of the interaction of EqtII with lipid membranes revealed that Chol is required for the penetration of the 30-residue-long extended N-terminal α-helix across the lipid bilayer to make a functional pore [117]. Given that Chol induces a negative curvature on the membrane, it is feasible that it also could facilitate pore formation by reducing the stress caused by membrane distortion. The results available indicate that, for an equivalent phase state, and SM and Chol content, these toxins preferred bilayer containing PC species whose acyl-chains consisted of 16 or 18 carbon atoms over those with shorter or longer acyl-chains [80]. An observation that correlated significantly with the length of the N-terminal α-helix responsible for membrane penetration, as stated above. How Chol may contribute to this behavior is still to be elucidated.

## 4. Perspectives and Final Remarks

Chol has profound influence on the function and biophysical properties of animal cell membranes. We still do not know the specific molecular basis that explains why Chol improves the actinoporin–membrane interaction, nor why these proteins show somewhat different behavior in the presence of this sterol. Fluorescence approaches with labeled lipid analogs can help in mapping the specific position of Chol molecules within the final pore assembly. These studies could be taken further with the use of cryo-electron microscopy to elucidate with atomic resolution the location of Chol, SM and other important lipids within the actinoporins’ protein–lipid pore complex. So far, only one crystalline structure, obtained in the absence of Chol, and involving the use of detergents, has been solved [14]. It has been recently proposed, based on molecular dynamics simulations, that, despite the specificity of actinoporins for SM that allows for membrane recognition, the aromatic loop thought to be responsible for SM binding would interact more with PC than SM after formation of thermodynamically stable pores [118]. The mentioned use of cryo-electron microscopy combined with membrane platforms such as nanodiscs, or even liposomes, with different lipid compositions would greatly help to solve where in the complex the different lipids are actually located.

Finally, actinoporins, and another similar pore-forming proteins, are increasingly becoming a matter of interest in bioengineering, to transform them into nanodevices with many different applications such as DNA or RNA sequencing [119,120], primary and tertiary structure determination of peptides and proteins [121,122], proteomic [31,123] and metabolomics studies [30], or green catalysts [124]. The influence of Chol on these promising biotechnological applications should not be neglected and, therefore, adds interest to our aim of understanding its unique role in actinoporins’ mode of action.

## Abbreviations

Chol	cholesterol
Eqt	equinatoxin
Fra	fragaceatoxin
OlyA	Ostreolysin A
OSM	Oleoyl-sphingomyelin
PC	phosphatidylcholine
PSM	palmitoyl-sphingomyelin
SM	sphingomyelin
Stns	sticholysins
